# 

**Published:** 1893-02-18

**Authors:** 


No. 334. Vol. XIII.] Saturday, February 18th, 1893. [Twopence.

